# RBBP6, a RING finger-domain E3 ubiquitin ligase, induces epithelial–mesenchymal transition and promotes metastasis of colorectal cancer

**DOI:** 10.1038/s41419-019-2070-7

**Published:** 2019-11-04

**Authors:** Chao Xiao, Gang Wu, Zhijie Zhou, Xin Zhang, YuPeng Wang, Guohe Song, Erxun Ding, Xing Sun, Lin Zhong, Shanbao Li, Junyong Weng, Zhonglin Zhu, Jian Chen, Xiaoliang Wang

**Affiliations:** 1 Department of General Surgery, Huashan Hospital, Fudan University, 20040 Shanghai, China; 2 Department of General Surgery, Henan Provincial People’s Hospital, People’s Hospital of Zhengzhou University, 450003 Zhengzhou, China; 3 Department of General Surgery, Shanghai General Hospital, School of Medicine, Shanghai Jiao Tong University, 200080 Shanghai, China; 4 Department of Liver Surgery, Liver Cancer Institute, Zhongshan Hospital, Fudan University, 200032 Shanghai, China; 5 Department of Thoracic Surgery, Shanghai Pulmonary Hospital, School of Medicine, Tongji University, 200433 Shanghai, China; 6 Department of General Surgery, Qingpu Branch of Zhongshan Hospital Affiliated to Fudan University, 201700 Shanghai, China

**Keywords:** Colon cancer, Metastasis

## Abstract

RBBP6 has been implicated in tumorigenesis but its role in tumor metastasis and progression has not been evaluated. Interestingly, here we show that RBBP6 is upregulated in colorectal cancer (CRC) where its expression level is positively correlated with distant metastasis. In this study, we identified RBBP6, a RING Finger-domain E3 ubiquitin ligase, served as an independent prognostic factor and predicted poor outcome for CRC patients. RBBP6 promoted cell proliferation, migration, and invasion in CRC cells and promoted tumor growth, lung metastasis, and liver metastasis in mouse models. Mechanistically, we revealed that RBBP6 bound and ubiquitylated IκBα, an inhibitor of the NF-κB-signaling pathway. RBBP6-mediated ubiquitination and degradation of IκBα significantly enhanced p65 nuclear translocation, which triggered the activation of NF-κB pathway and then induced the epithelial–mesenchymal transition (EMT) process and cell metastasis. Furthermore, by DNA methylation results and ChIP analysis, we demonstrated that the promoter of RBBP6 was hypomethylated, and was activated by multi-oncogenic transcription factors. In conclusion, our findings suggest that RBBP6 may be a potential prognostic biomarker and therapeutic target for CRC invasion and metastasis.

## Introduction

Colorectal cancer (CRC) is the third most commonly diagnosed cancer in the United States, accounting for more than 8% of all cancer-related deaths^1^. Despite new screening strategies and advances in treatment, distant metastasis remains the major obstacle to CRC therapy^2–4^. Approximately 20% of CRC patients will present with de novo metastatic disease and about 30% of stage II/III CRC patients will recur within 5 years after a curative intent surgery^5^. Moreover, the 5-year survival rate for patients diagnosed with distant metastatic disease is only 14.2%, but the rate for patients with localized CRC is 89.9%^6^. Therefore, it is critical to elucidate the molecular mechanisms that drive the process of CRC metastasis and to provide new therapeutic targets for CRC patients. Mounting evidence indicates that in epithelial cancers, including CRC, epithelial–mesenchymal transition (EMT) is constitutively activated, playing a tumor-promoting role by inducing tumor progression and metastasis^7,8^. During EMT, epithelial cells downregulate epithelial characteristics and acquire the mesenchymal phenotype, which increases the motility of individual cells and allows cancer cells to invade adjacent tissues and migrate to the distance^9,10^. A diversity of signaling pathways facilitate the initiation and progression of EMT, including Notch, transforming growth factor-β (TGF-β), WNT/β-catenin, RTK/Ras, ERK/MAPK, nuclear factor-κB (NF-κB) pathways, and others^11–13^.

The mammalian NF-κB subunit family includes five proteins: RELA (also known as p65), RELB, REL (also known as c-Rel), NF-κB1 (also known as p50/p105), and NF-κB2 (also known as p52/p100)^14^. NF-κB is composed of NF-κB1 or NF-κB2 bound to either RELA, RELB, or c-Rel. The most abundant form of NF-κB, p65/p50 dimer, which in its inactive state is kept in cytoplasm by the inhibitors of NF-κB (IκB)^15^. Generally, IκBs bind to p65/p50 dimer inhibiting their nuclear translocation and DNA binding. However, a variety of stimuli can activate the IκB kinase (IKK) complex, promoting the phosphorylation and ubiquitin-mediated degradation of IκBs, resulting in nuclear translocation of NF-κB and activation of the NF-κB-signaling pathway^16,17^. NF-κB has been reported constitutive activation in many kinds of human tumors, such as breast cancer^18^, ovarian cancer^19^, pancreatic cancer^20^, and gastric cancer^21^. There is growing evidence that NF-κB is also constitutively activated in CRC, playing the role of promoting tumor cell proliferation and inducing metastasis^22,23^. However, more research is needed to clarify the mechanism concerning NF-κB activation in CRC, which is one of the most aggressive types of cancer.

Retinoblastoma-binding protein 6 (RBBP6, also known as P2P-R or PACT) is a 200 kDa protein containing a conserved RING Finger domain, which functions as a negative regulator of tumor suppressors p53 and Rb^24^. As an E3 ubiquitin ligase, RBBP6 promotes the interaction between p53 and Hdm2, leading to Hdm2-mediated ubiquitination and degradation of p53^25^. Moreover, RBBP6 directly interact with multifunctional protein YB-1 through its RING Finger domain, leading to ubiquitination and proteosomal degradation of YB-1^26^. It also regulates DNA-replication and the stability of chromosomal common fragile sites (CFSs) via the RBBP6/ZBTB38/MCM10 axis^27^. RBBP6 is highly up-regulated and is correlated with poor clinical prognosis in a variety of cancers, such as esophageal cancer^28^, breast cancer^29^, lung cancer^30^, and cervical cancer^31^, playing a role in regulation of cell proliferation, cell cycle, and cell apoptosis. However, the biological function and expression pattern of RBBP6 in CRC remain poorly understood.

In this study, we compared the expression of RBBP6 in colorectal adenoma and matched adjacent normal tissues. Our results revealed a significant increase of RBBP6 in tumor tissues and the high expression of RBBP6 was closely related to poor survival of CRC patients. As an independent prognostic factor, high RBBP6 level was also related to histological grade, TNM stage, and distant metastasis in CRC patients. By bioinformatics analysis, we observed that the NF-κB-signaling pathway was strongly associated with the expression of RBBP6. Furthermore, RBBP6 promoted activating NF-κB-signaling pathway by ubiquitination and degradation of IκBα, the NF-κB inhibitor α. We further demonstrated that the promoter of RBBP6 was hypomethylated, and was bound and activated by multi-oncogenic transcription factor in CRC. Collectively, our investigation suggests that RBBP6 induces EMT and promotes metastasis of CRC via a mechanism involving NF-κB-signaling pathway.

## Results

### RBBP6 predicts poor prognosis in CRC and is involved in metastasis

**Fig. 1:**
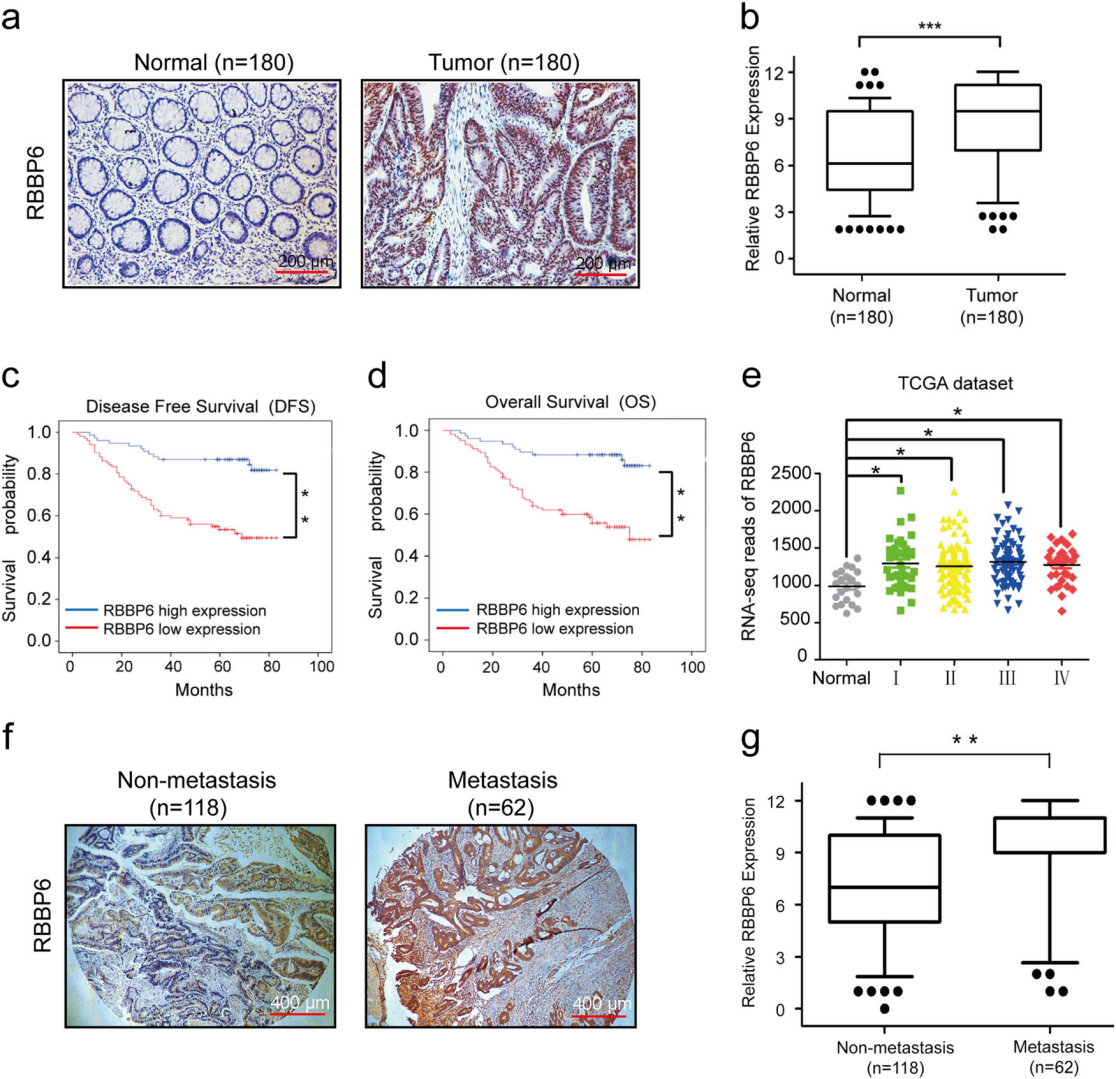
RBBP6 predicts poor prognosis in CRC and is involved in metastasis. **a** Representative expression levels of RBBP6 by immunohistochemistry performed with TMA of normal colon mucosa, primary cancer tissues from 180 patients. **b** Immunohistochemistry analysis of RBBP6 expression in normal mucosa and their matched tumor tissues. **c**, **d** Survival analysis showed that RBBP6 lower expression tumors have a favorable prognosis than RBBP6 higher expression tumors. **e** Expression of RBBP6 in normal specimens and colorectal carcinoma from TCGA dataset. **f**, **g** Immunohistochemistry analysis of RBBP6 expression in tumor without or with distant metastasis. *p *<* 0.05

To investigate the role of RBBP6 in CRC progression, we first analyzed the expression levels of protein RBBP6 in normal colon mucosae and matched tumor tissues using a tissue microarray containing 180 pairs of samples. The result showed that the positive rate of protein RBBP6 was significantly different between the tumor tissues and the adjacent normal colon tissues (Fig. 1a and Table 1). RBBP6 was highly expressed in 103 of 180 (57.2%) colon tumor tissues compared with 62 of 180 (34.4%) adjacent normal mucosae tissues (Fig. 1b). By western blotting, we revealed that RBBP6 was markedly overexpressed in eight primary CRC samples compared with the matched adjacent normal tissues (Supplementary Fig. 1A). Moreover, patients with a high RBBP6 expression signature possessed a shorter disease-free survival (DFS) time and overall survival (OS) time than those with a low RBBP6 expression by Kaplan–Meier analysis (*P* < 0.01; Fig. 1c, d). TCGA database (Fig. 1e) and GEO database (Supplementary Fig. 1B) also showed RBBP6 levels were significantly upregulated in tumor tissues compared with normal tissues. In addition, the expression levels of RBBP6 were correlated with histological grade (*P* = 0.036), TNM stage (*P* = 0.006), and distant metastasis (*P* = 0.018; Table 2). Cox regression analyses demonstrated that RBBP6 was an independent predictor for CRC patients (Supplementary Fig. 1C). These results indicate that RBBP6 overexpression may predict poor prognosis in CRC.

**Table 1:** Expression of RBBP6, p-p65, N-cadherin, and E-cadherin in 180 cases of colorectal cancer and adjacent normal mucosa tissues (*χ*^2^-test)

Proteins	CRC tissues (*n* = 180)	%	Adjacent normal mucosae (*n* = 180)	%	*P*-value
*RBBP6*					
High	103	57.2	62	34.4	<0.001
Low	77	42.8	118	65.6	
*p-p65*					
High	109	60.6	84	46.7	0.011
Low	71	39.4	96	53.3	
*E-cadherin*					
High	68	37.8	98	54.4	0.002
Low	112	62.2	82	45.6	
*N-cadherin*					
High	106	58.9	78	43.3	0.004
Low	74	41.1	102	56.7

**Table 2:** Correlation between clinicopathologic features and the expression of RBBP6, p-p65, N-cadherin, and E-cadherin in 180 cases of colorectal cancer tissues (*χ*^2^-test)

Variable	Number	RBBP6			p-p65			N-cadherin			E-cadherin		
		High	low	*P*	High	low	*P*	High	low	*P*	High	low	*P*
*Age*		103	77		109	71		106	74		68	112	
<65	64	34	30	0.434	36	28	0.427	34	30	0.27	26	38	0.631
≥65	116	69	47		73	43		72	44		42	74	
*Gender*													
Male	93	51	42	0.548	54	39	0.542	52	41	0.45	32	61	0.359
Female	87	52	35		55	32		54	33		36	51	
*Tumor size*													
<5 cm	96	53	43	0.651	60	36	0.647	53	43	0.293	32	64	0.219
≥5 cm	84	50	34		49	35		53	31		36	48	
*Histological grade*													
Well, moderate	82	40	42	0.036	43	39	0.047	41	41	0.034	38	44	0.032
Poor	98	63	35		66	32		65	33		30	68	
*TNM stage*													
I–II	79	36	43	0.006	40	39	0.021	39	40	0.023	38	41	0.013
III–IV	101	67	34		69	32		67	34		30	71	
*Distant metastasis*													
M0	118	60	58	0.018	64	54	0.024	62	56	0.025	48	70	0.332
M1	62	43	19		45	17		44	18		20	42	

*RBBP6* Retinoblastoma-binding protein 6, *TNM* tumor node metastasis

By analysis of the tissue microarray containing 180 pairs of samples, we found 43 of 62 (69.4%) CRC patients with distant metastasis but only 60 of 118 (50.8%) CRC patients without distant metastasis had high RBBP6 expression (Table 2 and Fig. 1f, g). Interestingly, RBBP6 was also highly expressed in highly invasive cancer cells (SW620, Caco2, HT29, LoVo) compared with low-invasive cancer cells (RKO, SW480, HCT15) (Supplementary Fig. 1D). Taken together, these results suggest that RBBP6 upregulation is relevant to metastasis of CRC.

### RBBP6 promotes CRC cell proliferative, migratory, and invasive capacity in vitro

To investigate the oncogenic activity of RBBP6 in CRCs, we retrovirally established overexpression of RBBP6 in RKO and SW480 cells (designated as RKO-RBBP6 and SW480-RBBP6) in which RBBP6 expression level was lower than other cell lines, and silenced RBBP6 in HT29 and SW620 cells (designated as HT29-sh-RBBP6 and SW620-sh-RBBP6) in which RBBP6 expression level was higher than other cell lines.

**Fig. 2:**
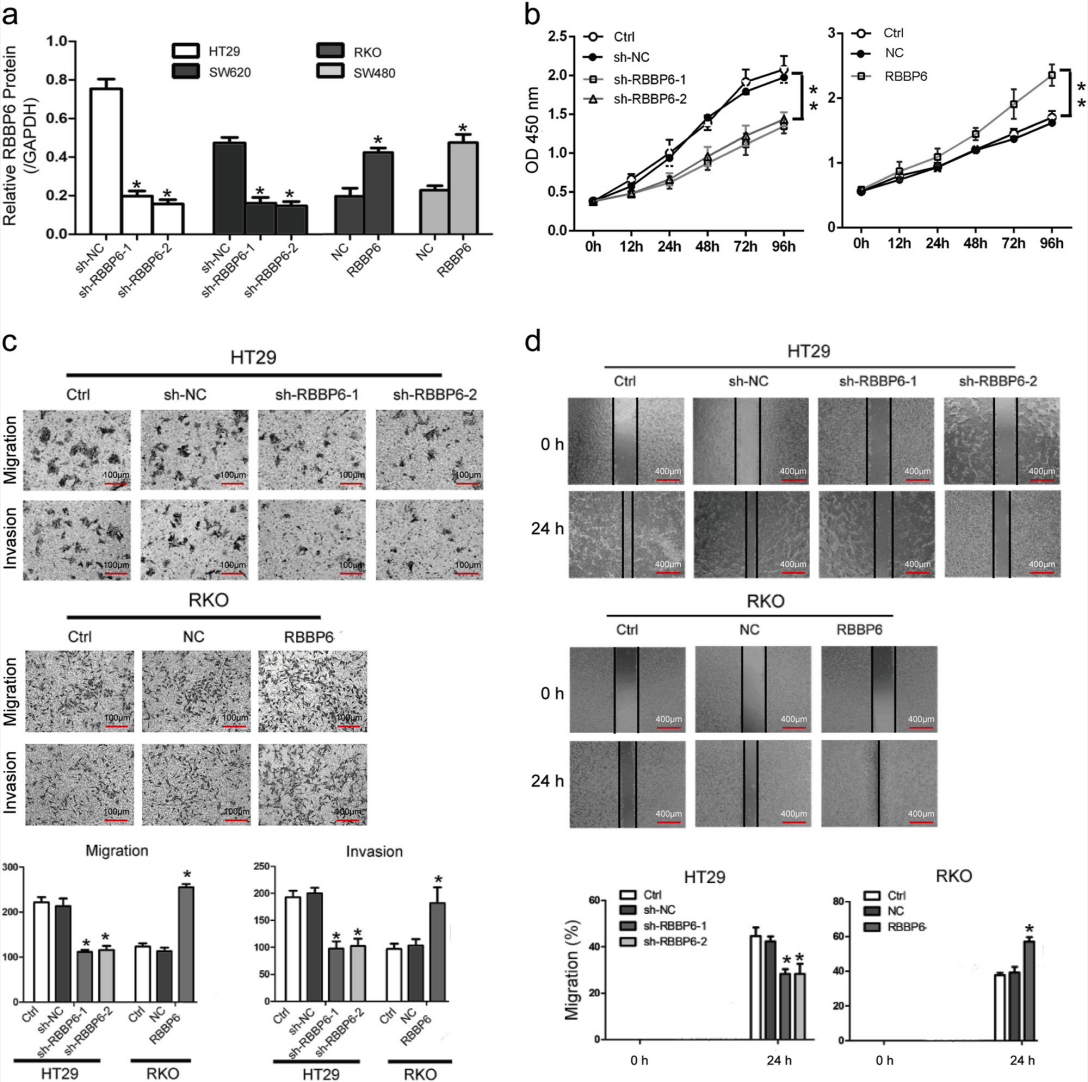
RBBP6 promotes CRC cell proliferative, migratory, and invasive capacity in vitro. **a** The RBBP6 knockdown and overexpression effects were confirmed by Western blot. **b** Cell proliferation rates were measured by CCK8 assay. The results were measured at an optical density of 450 nm. **c**, **d** Cell motility were analyzed by (**c**) wound healing assay, (**d**) transwell migration and matrigel invasion assays. Data represent three independent experiments. Data are means ± SD. *p *<* 0.05

The expression levels of RBBP6 in the resultant cell lines were confirmed by western blotting (Fig. 2a; Supplementary Fig. 2A). Then, we performed CCK-8 assay to examine the role of RBBP6 in CRC cells growth. Compared to RKO-NC (negative control cells) and SW480-NC cells, the ability of both RKO-RBBP6 and SW480-RBBP6 cells in cell proliferation has been improved significantly. In contrast, silencing RBBP6 in HT29 and SW620 cells significantly inhibited cell proliferation (Fig. 2b; Supplementary Fig. 2B). These results suggest that RBBP6 is a crucial regulator of proliferation in CRC cells.

We further assessed the metastatic ability of CRC cells with different levels of RBBP6 expression. The transwell migration assay and invasion assay indicated that both RKO-RBBP6 and SW480-RBBP6 cells showed a higher invasive rate compared with their control cells. In contrast, silencing RBBP6 significantly reduced the migration and invasion of HT29 and SW620 cells (Fig. 2c; Supplementary Fig. 2C). Moreover, the result was verified by wound-healing assay, both RKO-RBBP6 and SW480-RBBP6 cells showed significantly faster closure of wound area compared to respective control cells (Fig. 2d; Supplementary Fig. 2D). The results indicate that RBBP6 promotes the CRC cell migratory and invasive capacity in vitro.

### RBBP6 promotes CRC cell proliferation and metastasis in vivo

**Fig. 3:**
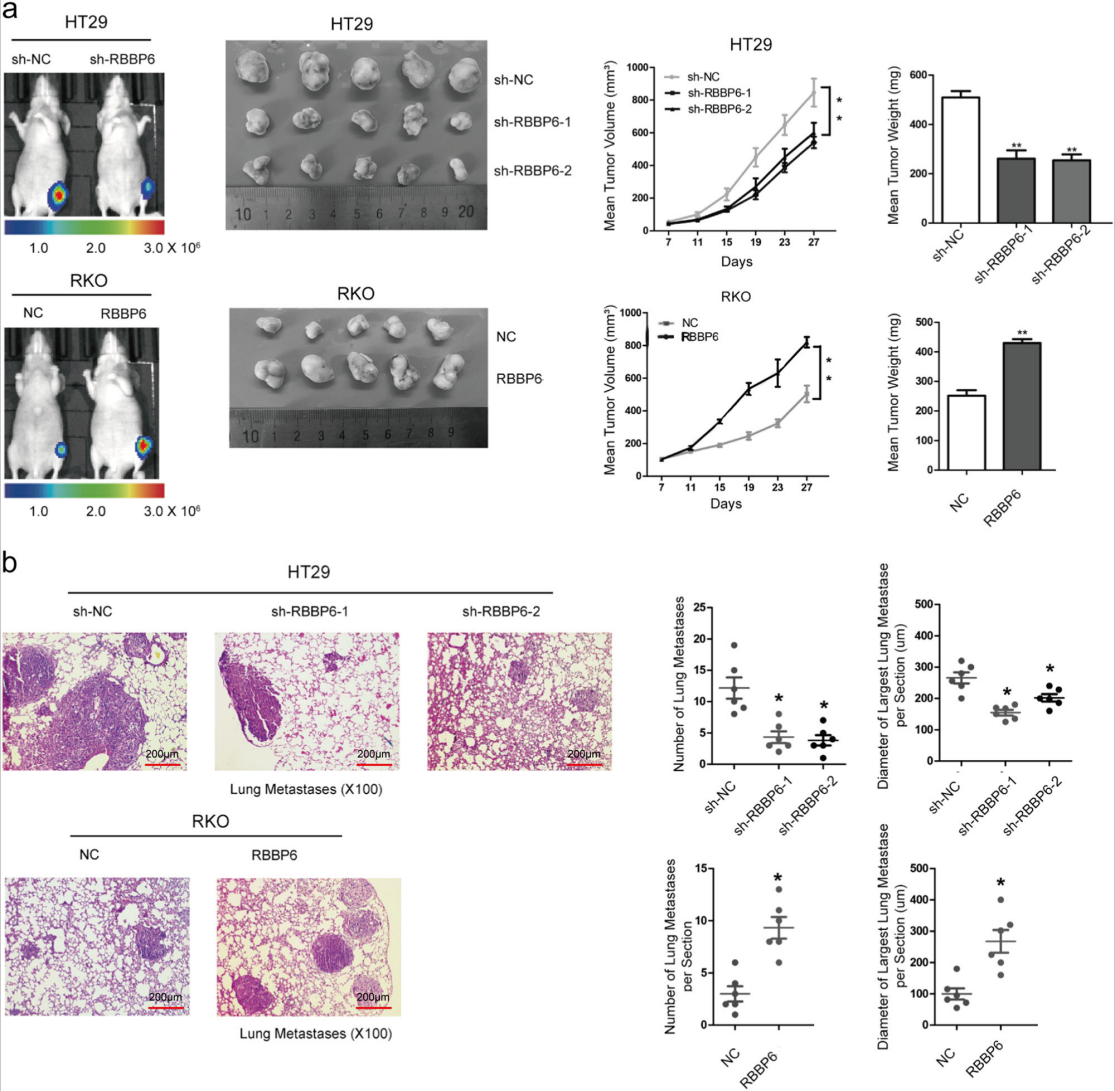
RBBP6 promotes CRC cell proliferation and metastasis in vivo. **a** Stable HT29 and RKO cells were subcutaneously injected into the groins of nude mice: Representative images of tumor-bearing mice and tumors from mice in each group (left); tumor volumes and tumor weights were measured on the indicated days (right). **b** Lung metastasis model was constructed: representative images of mice intravenously injected with stable cells and H&E staining of the metastatic nodules in the lung (left); the numbers and diameters of metastatic nodules in the lungs of mice (right). Data are means ± SD. *p *<* 0.05

To investigate whether RBBP6 could enhance proliferative capacity of CRC cells in vivo, RKO-RBBP6, HT29-sh-RBBP6 cells, and their control cells were subcutaneously injected into the groins of nude mice. As expected, the tumors from RKO-RBBP6 and HT29-sh-NC cells grew faster and larger than those from their respective control cells (Fig. 3a).

To further study the effect of RBBP6 on metastasis of CRC in vivo, modified RKO and HT29 cells were injected into nude mice via the tail vein to develop a lung metastasis model. As the result showed, RBBP6 overexpression not only dramatically increased the number of lung metastatic tumors, but also remarkably increased the average diameter of the metastatic tumors (Fig. 3b).

In addition, RKO and HT29 cells were transplanted into the spleens of nude mice to construct a liver metastasis model. Similarly, a significant difference was found in the number and size of liver metastatic tumors between RKO-RBBP6 or HT29-sh-NC cells and their control cells (Supplementary Fig. 3).

### RBBP6 promotes CRC cell motility by modulating EMT

**Fig. 4:**
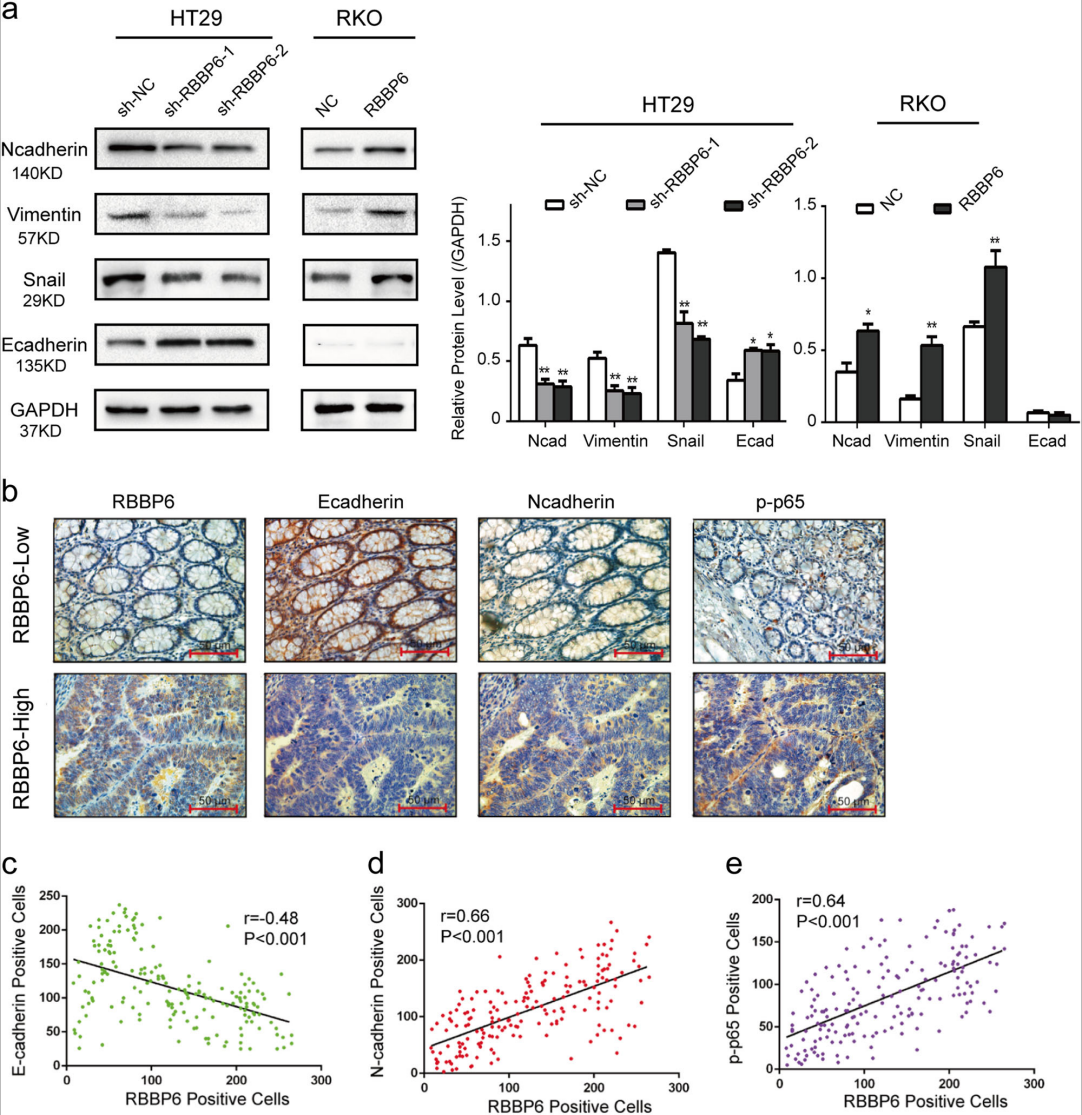
RBBP6 promotes CRC cell motility by modulating EMT. **a** Expression of mesenchymal markers (N-cadherin, Vimentin, and Snail) and epithelial markers (E-cadherin) in CRC cells was analyzed by Western blot and Immunofluorescence assay. **b** Representative IHC staining of E-cadherin, N-cadherin, and p-p65 in CRC tumor tissues with low or high RBBP6 expression. **c**–**e** Spearman’s correlation analyses showed that RBBP6 was positively associated with N-cadherin and p-p65 expression, but negatively correlated with E-cadherin expression in 180 CRC tumor tissues. Data are means ± SD. *p *<* 0.05

In view of the fact that EMT is a critical event in tumor invasion and metastasis, we assessed whether EMT was responsible for RBBP6-mediated changes in CRC cell motility. Western blotting confirmed increased expression of mesenchymal markers (N-cadherin, Vimentin, and Snail) and decreased expression of epithelial markers (E-cadherin) in RKO-RBBP6, HT29-sh-NC, SW480-RBBP6, SW620-sh-NC cells compared to their respective control cells (Fig. 4a; Supplementary Fig. 4A). Moreover, expression changes of epithelial and mesenchymal markers were also confirmed by western blotting and immunohistochemistry analysis performed with subcutaneous tumor tissues derived from nude mice (Supplementary Fig. 4B and C), suggesting that RBBP6 enhances EMT in vivo.

We further analyzed the correlation between the expression of RBBP6 and the EMT-related proteins (E-cadherin, N-cadherin) in 180 samples (Fig. 4b). The results showed that the expression of E-cadherin was negatively correlated with the expression of RBBP6 (*P* < 0.001, *r* = −0.48; Fig. 4c). The expression of N-cadherin was positively correlated with the expression of RBBP6 (*P* < 0.001, *r* = 0.66; Fig. 4d). These results demonstrate that RBBP6 promotes CRC cell motility by inducing EMT.

### RBBP6 promotes the activation of NF-κB-signaling pathway in CRC

**Fig. 5:**
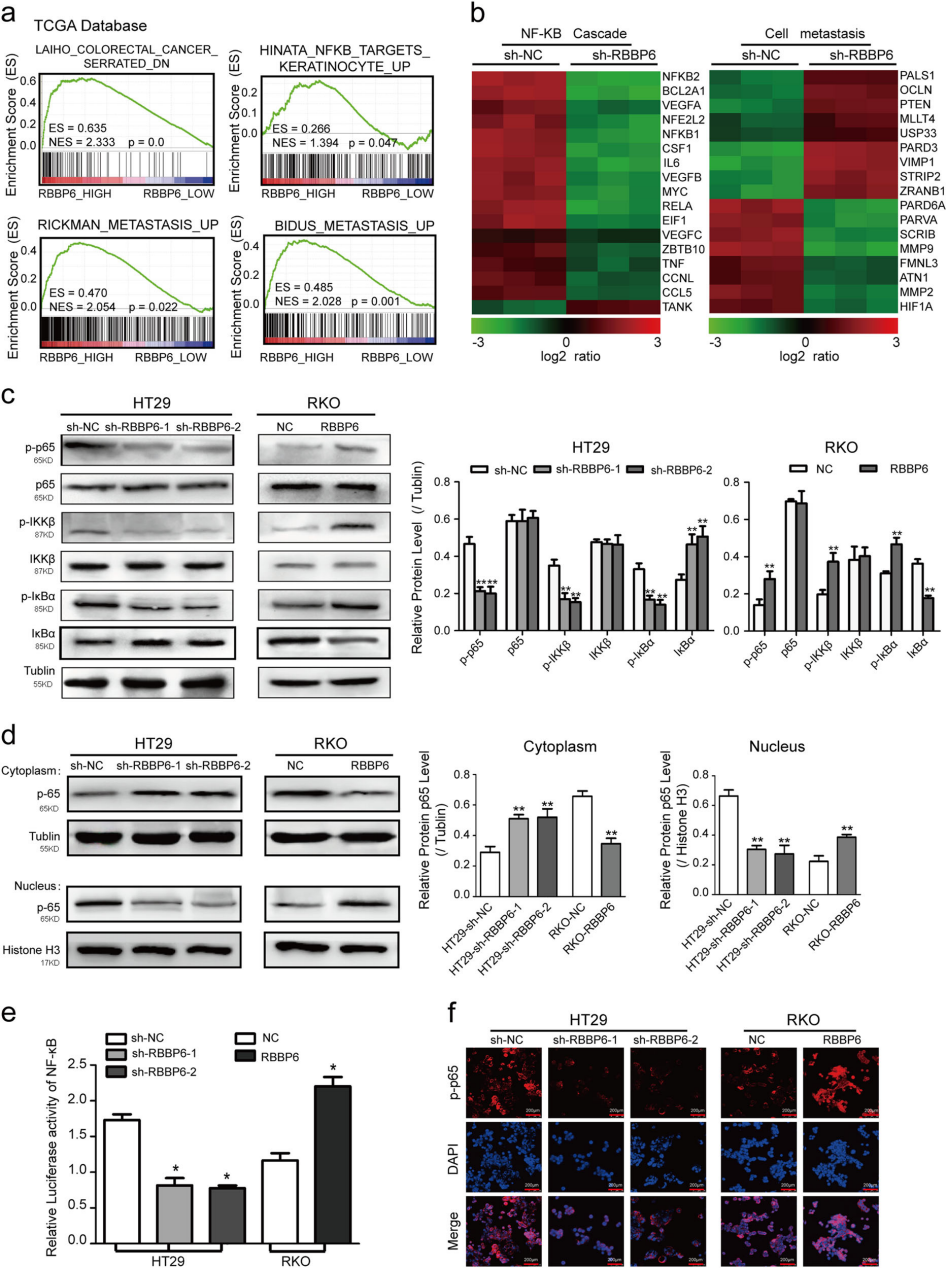
RBBP6 promotes the activation of NF-κB-signaling pathway in CRC. **a** GSEA comparing RBBP6 higher expression group (red) against RBBP6 lower expression group (blue) of patients with CRC in the TCGA dataset: the level of RBBP6 mRNA was positively correlated with CRC metastasis and the NF-κB-activated gene signatures. **b** Heatmaps showing the representative GO terms enriched within RBBP6-regulated genes. **c** Proteins involved in the NF-κB-signaling pathway were determined by Western blotting in HT29 and RKO cells. **d** Levels of p65 in nuclear and cytoplasmic extracts were measured by Western blotting in HT29 and RKO cells. **e** Luciferase reporter assay showing NF-κB transcriptional activity in HT29 and RKO cells. **f** Representative immunofluorescent staining showing the nuclear translocation of p-p65 in HT29 and RKO cells

To better understand the biologic pathways by which RBBP6 involved in CRC pathogenesis and progression, we performed gene set enrichment analysis (GSEA) in TCGA database. The data indicated that RBBP6 expression was positively correlated with CRC development, neoplasm metastasis, and NF-κB-signaling gene signatures (Fig. 5a). Furthermore, gene expression profiling was performed on HT29-sh-RBBP6 and its control cells. As the microarray analysis showed, the expression of NF-κB downstream genes and cell metastasis-related genes is different significantly (Fig. 5b), supporting the possibility that RBBP6 promotes CRC metastasis via NF-κB-signaling pathway. Consistent with this supposition, western blotting revealed that RBBP6 overexpression significantly elevated the phosphorylated levels of p65, IKKβ, and IκBα, whereas p-p65, p-IKKβ, and p-IκBα were reduced in RBBP6-silenced cells (Fig. 5c; Supplementary Fig. 5A). Moreover, the nuclear translocation of p65 were enhanced in RKO-RBBP6 and HT29-sh-NC (Fig. 5d, f) or SW480-RBBP6 and SW620-sh-NC cells (Supplementary Fig. 5B). The NF-κB luciferase reporter activity was dramatically decreased in HT29-sh-RBBP6 cells but increased in RKO-RBBP6 cells compared to their respective control cells (Fig. 5e). By the immunohistochemistry, we also observed that the expression level of RBBP6 is positively related to the expression level of p-p65 (*P* < 0.001, *r* = 0.64; Fig. 4e). Collectively, these results suggest that RBBP6 promotes the activation of NF-κB-signaling pathway.

### RBBP6 regulates the ubiquitination of IκBα to activate NF-κB-signaling pathway

**Fig. 6:**
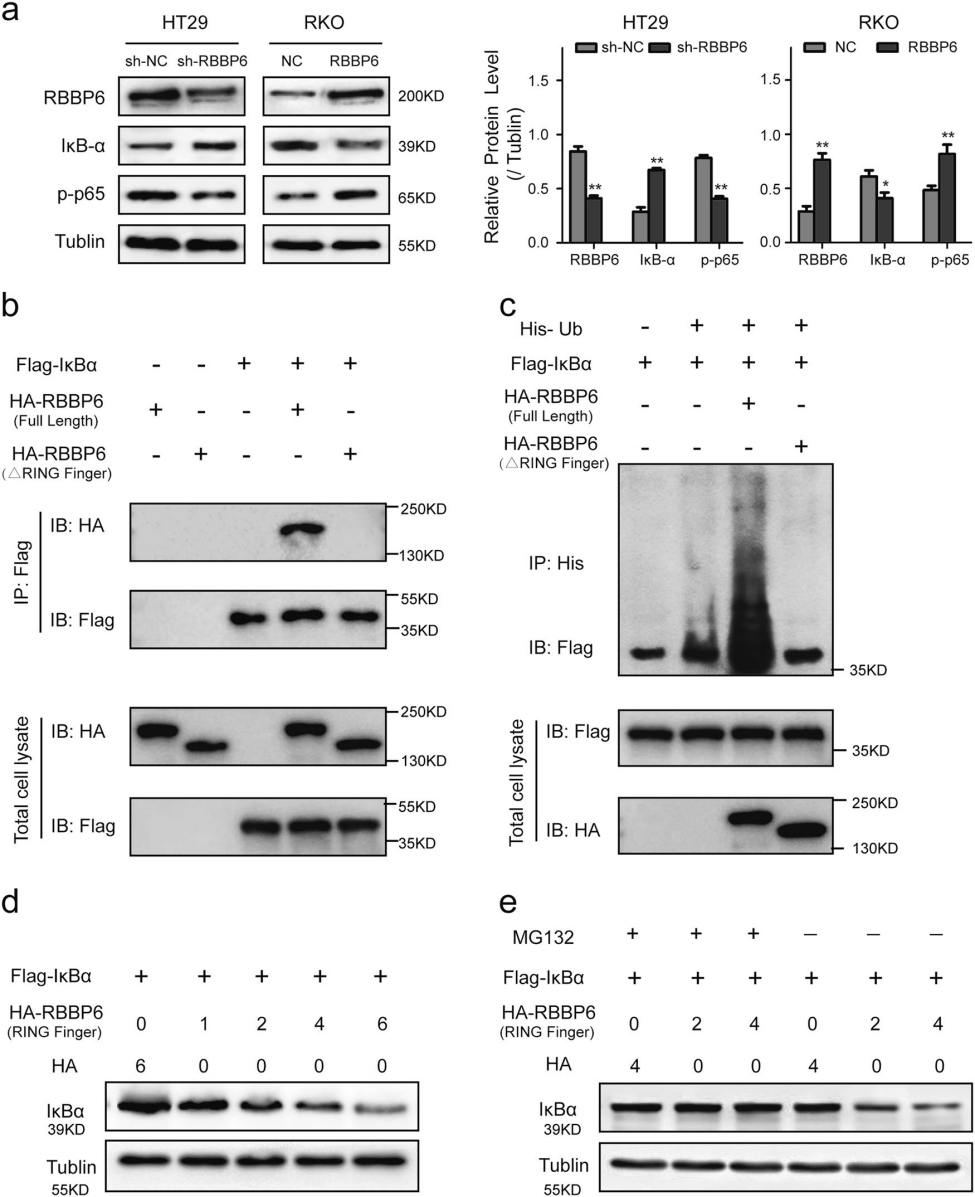
RBBP6 regulates the ubiquitination of IκBα to activate NF-κB-signaling pathway. **a** Western blotting showing the expression of RBBP6, IκBα, and p-p65: RBBP6 suppressed IκBα protein expression and promoted p-p65 protein expression in CRC cells. **b** Immunoprecipitation (IP) demonstrated RBBP6 interacted with IκBα through the RING Finger domain: HEK293 cells were co-transfected with plasmids expressing Flag-IκBα and HA-RBBP6 (full length) or HA-RBBP6 (ΔRing Finger) lacking the Ring Finger domain. **c** The Ring Finger domain of RBBP6 is required for ubiquitination of IκBα in vivo: HEK293 cells were co-transfected with plasmids expressing His-Ub, Flag-IκBα, and HA-RBBP6 (full length) or HA-RBBP6 (ΔRing Finger), co-immunoprecipitation revealed the polyubiquitination of Flag-IκBα. **d** Exogenously expressed HA-Ring Finger reduced the levels of exogenously expressed Flag-IκBα in a dose-dependent manner in HEK293 cells. **e** MG132 partly restored the IκBα protein levels that were reduced by the HA-RING Finger plasmid

RBBP6 was observed to activate NF-κB-signaling pathway, therefore, we further investigated the mechanism that RBBP6 activates the pathway. Firstly, Western blotting showed that silencing of RBBP6 upregulated protein IκBα (the NF-κB inhibitor α) level and suppressed the expression of p-p65 in HT29 cells. Conversely, overexpression of RBBP6 had the opposite effect in RKO cells (Fig. 6a). To further study the interaction between RBBP6 and IκBα protein within the cell, HEK293 cells were co-transfected with plasmids expressing Flag-IκBα and HA-RBBP6 (full length) or HA-RBBP6 (ΔRing Finger) lacking the Ring Finger domain. By immunoprecipitation and Western blot analysis, we found that Flag-IκBα was able to precipitate full-length HA-RBBP6, However, Flag-IκBα was not able to precipitate ΔRing-Finger HA-RBBP6 that lacks the Ring Finger domain (Fig. 6b).

Moreover, the Ring Finger domain of RBBP6 is required for IκBα ubiquitination. As the result showed, full-length HA-RBBP6 significantly enhanced IκBα ubiquitination, whereas the ΔRing-Finger mutant of HA-RBBP6 hardly had any effect on IκBα ubiquitination (Fig. 6c). Modification of proteins by ubiquitin may result in proteosomal degradation. Therefore, we investigated whether IκBα could be degraded by the proteosome as a result of ubiquitination by the RING Finger domain of RBBP6. We co-transfected HEK293 cells with Flag-IκBα plasmid and increasing amounts of HA-RING Finger plasmid. The result showed that exogenously expressed HA-Ring Finger reduced the levels of exogenously expressed Flag-IκBα in a dose-dependent manner (Fig. 6d). However, MG132, a proteasome inhibitor, partly restored the levels of IκBα protein that were reduced by the HA-RING Finger plasmid (Fig. 6e). Taken together, these results reveal that RBBP6 might promote the proteasome-dependent degradation of ubiquitinated IκBα, and then activate the NF-κB-signaling pathway.

### The NF-κB-signaling pathway are involved in RBBP6-induced EMT and metastasis

To further determine whether NF-κB is the central signaling pathway by which RBBP6 promotes the EMT process in CRC, we treated the RKO-RBBP6 cells with exogenous IκBα-M (IκBα dominant-negative mutant, which can block NF-κB activation but cannot be ubiquitinated and degraded by proteasome) or NF-κB inhibitor BAY11-7082, and treated the HT29-sh-RBBP6 cells with plasmid expressing p65 which can promote the activation of NF-κB pathway.

**Fig. 7:**
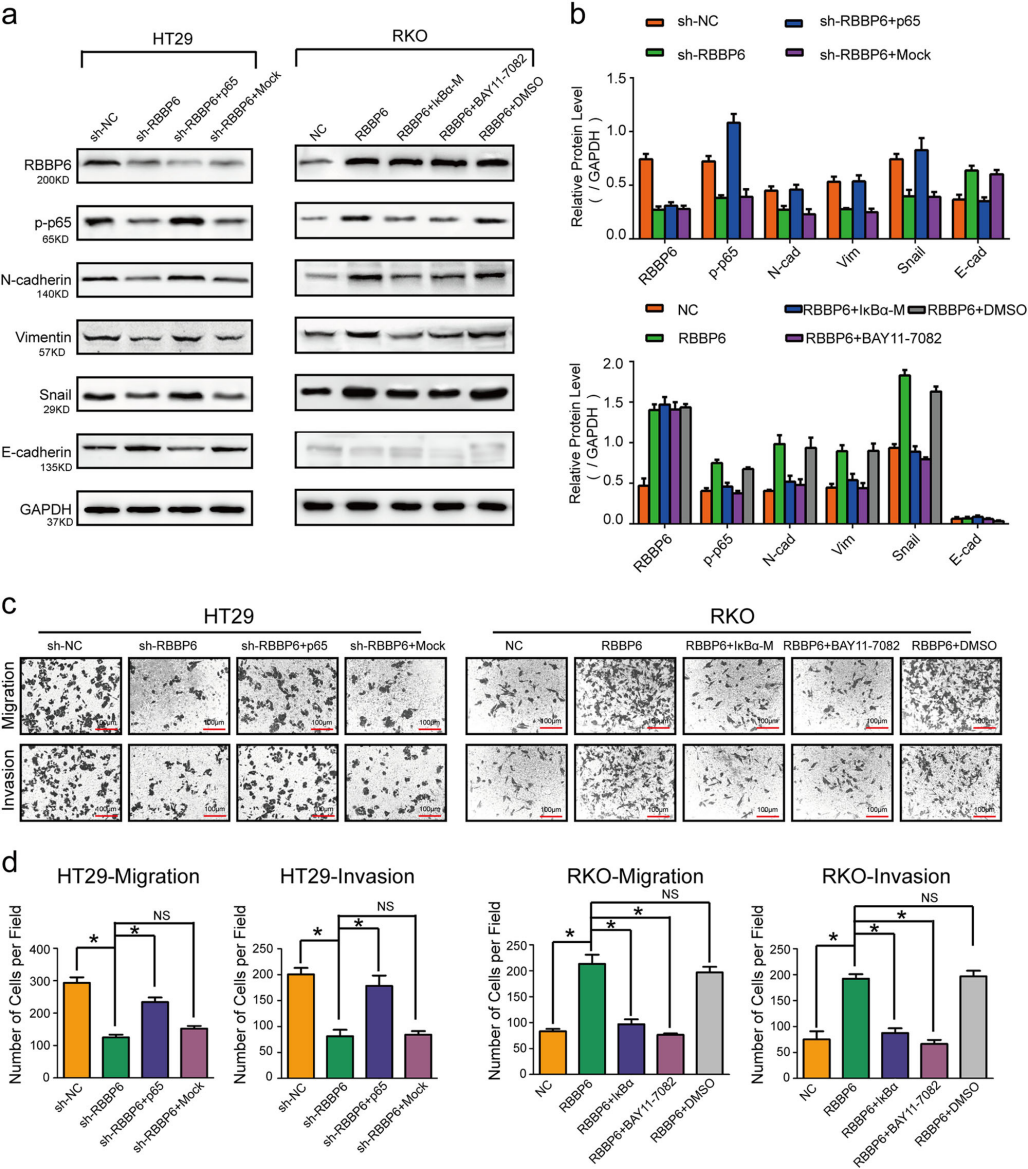
The NF-κB-signaling pathway are involved in RBBP6-induced EMT and metastasis. **a**, **b** Expression of mesenchymal markers (N-cadherin, Vimentin, and Snail) and epithelial markers (E-cadherin) in CRC cells transfected with plasmids expressing IκBα-M, p65, or treated with NF-κB inhibitor BAY11-7082. **c**, **d** Transwell migration and matrigel invasion assays of HT29 and RKO cells

As the result showed, western blotting confirmed that either exogenous IκBα-M or NF-κB inhibitor BAY11-7082 treatment decreased expression of mesenchymal markers (N-cadherin, Vimentin, and Snail) and increased expression of epithelial markers (E-cadherin) in RKO-RBBP6 cells, whereas the overexpression of p65 in HT29-sh-RBBP6 cells increased expression of mesenchymal markers and decreased expression of epithelial markers (Fig. 7a, b). Correspondingly, either exogenous IκBα-M or NF-κB inhibitor BAY11-7082 treatment dramatically abolished the effects of RBBP6-overexpression on CRC cell migration and invasion, whereas the overexpression of p65 restored the effects of RBBP6-sh on CRC cell migration and invasion (Fig. 7c, d; Supplementary Fig. 6).

### Mechanism underlying RBBP6 overexpression in CRC

**Fig. 8:**
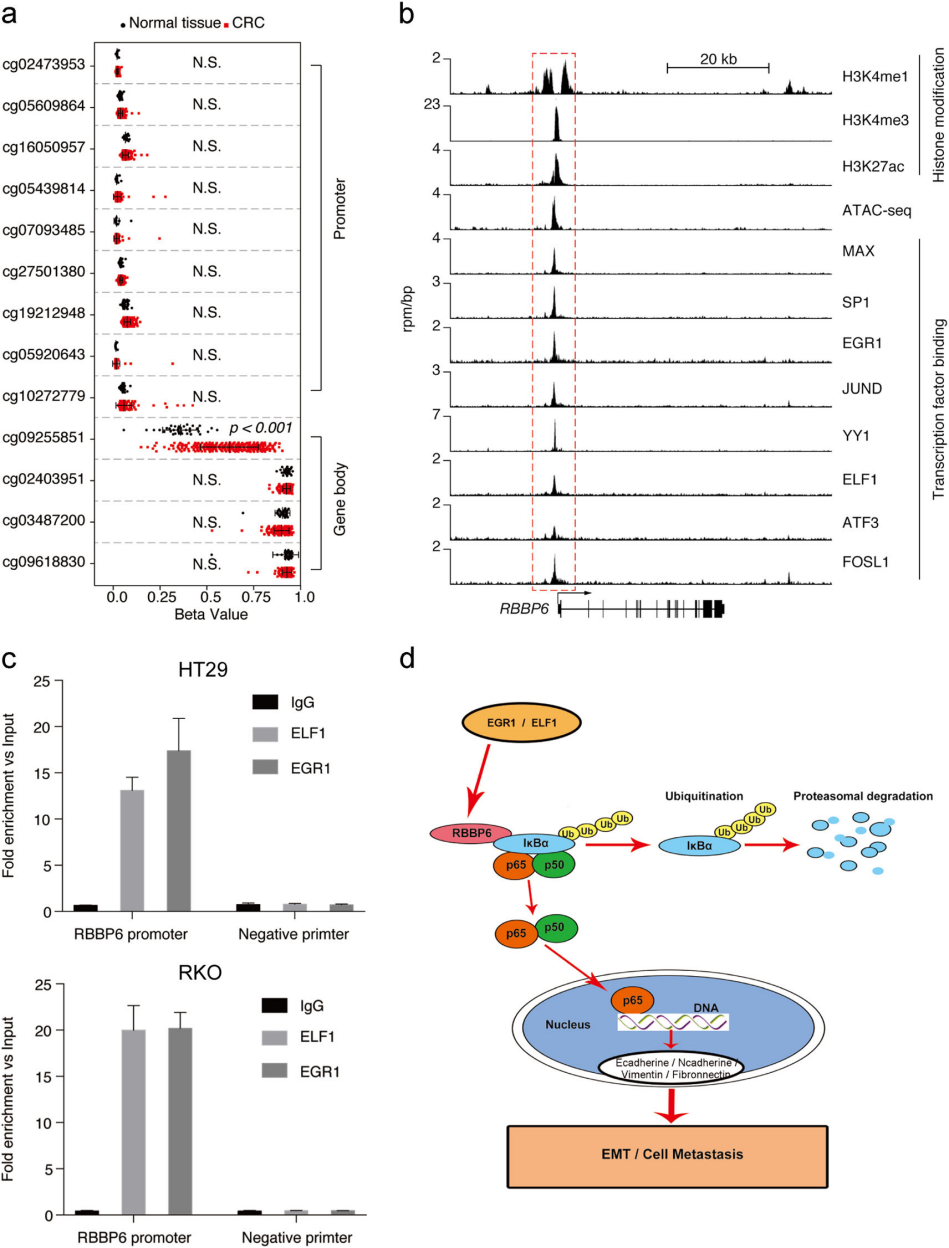
Hypomethylation and binding by multi-oncogenic transcription factor on promoter of RBBP6 as well as hypermethylation on gene body contribute to RBBP6 overexpression in CRC. **a** Analysis of DNA methylation of RBBP6 in CRC. DNA methylation was determined by hm450 results from TCGA database. Beta value of each probe around RBBP6 promoter and gene body regions in CRC and normal tissue were plotted. **b** Analysis of the regulatory regions of RBBP6 using ChIP-seq database in GEO database. **c** Validation of ELF1 and EGR1 binding on the RBBP6 promoter. ChIP-qPCR was performed using antibodies against ELF1 and EGR1. Enrichment of ELF1 and EGR1 on RBBP6 was normalized with input. Normal IgG was used as negative control. **d** The model showing roles of RBBP6 during EMT process

To investigate the mechanism underlying RBBP6 over amplification in CRC, we first performed DNA methylation analysis on the promoter and gene body of RBBP6 using the hm450 results from TCGA database^32^. We found that the DNA methylation on the promoter of RBBP6 was similar in colorectal tumor and normal colon tissue, but the DNA methylation on the first intron of RBBP6 was higher in CRC comparing to normal colon tissue (Fig. 8a). Emerging evidence has demonstrated that unlike methylation in the promoter, DNA methylation on gene body might stimulate transcription elongation and thus leads to gene activation^33^. These results suggested that DNA methylation on the first intron of RBBP6 might contribute to RBBP6 overexpression in CRC.

To further investigate the cis-element that contribute to RBBP6 overexpression, we retrieved the GEO database available ChIP-seq data in CRC cell lines^34,35^. We first identified that the active promoter/enhancer of the RBBP6 by using the H3K4me3 and H3K27ac ChIP-seq datasets (Fig. 8a). We then found that the oncogenic transcription factor including MAX, SP1, EGR1, JUND, YY1, ELF1, ATF3, and FOSL1 were all bound at the promoter region of RBBP6, suggesting that the RBBP6 was activated by the multi-oncogenic transcription factor and all of these oncogenic transcription factors might contribute to RBBP6 activation in CRC (Fig. 8b). To validate this, we performed ChIP-qPCR using antibodies against EGR1, ELF1 in HT29 and RKO cells. Consistent with the ChIP-seq experiments, EGR1 and ELF1 were both bound at the promoter regions of RBBP6 in HT29 and RKO cells (Fig. 8c). In summary, DNA methylation results and ChIP analysis demonstrated that the promoter of RBBP6 was hypomethylated and was bound and activated by the multi-oncogenic transcription factor in CRC. Taken together, these results demonstrate that RBBP6 promotes the EMT process by activating the NF-κB-signaling pathway via ubiquitination of IκBα in CRC (Fig. 8d).

## Discussion

In present study, we revealed for the first time that RBBP6 promotes the invasion and metastasis of CRC by regulating the NF-κB-signaling pathway. We compared RBBP6 expression between CRC tissues and adjacent normal tissues in relation to CRC pathogenesis in TCGA dataset, GEO dataset and 180 colon cancer patients from Shanghai General Hospital. RBBP6 was markedly upregulated in CRC tissues compared with the normal mucosae tissues and correlated with a poor prognosis of CRC patients. These results are consistent with our previous study that RBBP6 overexpression was associated with a poor survival rate in colon cancer^36^. Most importantly, IHC analysis demonstrated that the level of RBBP6 expression was much higher in tumors with distant metastasis than that in tumors with no distant metastasis. RBBP6 displayed a significant upregulation in highly invasive CRC cells compared with low invasive cells. Moreover, RBBP6 overexpression was found to be positively correlated with histological grade, TNM stage and distant metastasis in CRC patients. In addition, RBBP6 overexpression in cultured CRC cells significantly promoted proliferation, migration, invasion, EMT in vitro, and enhanced metastatic capacity in vivo by developing xenograft mouse models, while downregulation of RBBP6 produced the opposite effects. These findings suggest that RBBP6 may play an important role in promoting progression and metastasis of CRC.

Metastasis is a multistep and complex process by which tumor cells disseminate from the primary site to the distant tissue and form secondary tumors^37^. In CRC, tumor cells lose the epithelial phenotype and gain the mesenchyme-like phenotype which results in increased invasiveness, thereby initiating the early step of the metastatic cascade^38^. In this study, we observed that RBBP6 overexpression upregulated the levels of N-cadherin, Vimentin, Snail, and downregulated the levels of E-cadherin, whereas RBBP6 inhibition had the opposite effects. These findings suggest that RBBP6 promotes carcinogenesis and progression of CRC via modulating EMT process. Multiple signaling pathways involved in the initiation and progression of EMT^12^. Accumulated evidence has revealed that NF-κB is constitutively expressed in both CRC cell lines and CRC tissues^39,40^. NF-κB induces EMT by activating transcription factors, such as Twist or Snail, playing a pivotal role in tumor metastasis^41^. In present study, the bioinformatics analysis showed that NF-κB were positively correlated with alterations of RBBP6 in CRC. We confirmed that RBBP6 increased the phosphorylation of p65 and promoted p65 translocation into the nucleus, without changing the total levels of p65. Furthermore, exogenous IκBα-M or NF-κB inhibitor treatment dramatically abolished the effects of RBBP6 on EMT, cell proliferation, migration, and invasion. These results demonstrate that RBBP6 promotes the EMT process by activating the NF-κB-signaling pathway in CRC.

RBBP6 has been proved to be highly expressed in a variety of cancers, including those of the esophagus^28^, breast^29^, lung^30^, and cervix^31^. Moreover, the RBBP6 protein can interact with multiple proteins. It is a multi-domain protein containing Rb-binding and p53-binding domains together with RING Finger and DWNN domains^42,43^, which are related to protein degradation function. RBBP6 can act as a scaffold on which p53 interacts with Hdm2, leading to Hdm2-mediated ubiquitination and degradation of p53^25^. As an E3 ligase, RBBP6 directly interact with YB-1 through the RING Finger domain, leading to ubiquitination and degradation of YB-1^26^. It also regulates DNA-replication and the stability of chromosomal CFSs via ubiquitination and proteasomal degradation of ZBTB38^27^. Interestingly, we observed that RBBP6 could reduce the expression of IκBα protein, an inhibitor of NF-κB-signaling pathway. In fact, Co-immunoprecipitation analysis showed that RBBP6 bound to IκBα through the RING Finger domain. RBBP6 also induced the polyubiquitylation of IκBα and might involve in the proteasome-dependent degradation of IκBα. Therefore, we deduced that RBBP6 could promote activating NF-κB-signaling pathway by ubiquitination and degradation of IκBα, and further induce EMT and metastasis in CRC. However, more study is needed to clarify the mechanism by which RBBP6 increases in a variety of cancers. By DNA methylation results and ChIP analysis, we found that the oncogenic transcription factor including MAX, SP1, EGR1, JUND, YY1, ELF1, ATF3, and FOSL1 were all bound at the promoter region of RBBP6. We demonstrated the promoter of RBBP6 was hypomethylated and was bound and activated by the multi-oncogenic transcription factor in CRC.

In summary, we reported that RBBP6 expression was significantly upregulated in CRC, and RBBP6 overexpression was correlated with shorter OS of CRC patients. Overexpression of RBBP6 promotes EMT and metastasis in CRC by activating the NF-κB pathway via ubiquitination of IκBα. Our findings suggest that RBBP6 plays a pivotal role in the progression of colorectal tumorigenesis and it may be a potential prognostic biomarker and therapeutic target for CRC invasion and metastasis.

## Materials and methods

### Patient specimens and immunohistochemistry

A total of 180 patients who had surgery for CRC between January 2006 and December 2010 at Shanghai General Hospital were selected for the present study. No patients had ever received chemotherapy or radiotherapy before the surgery. Tissue microarrays (TMA) including the 180 primary colorectal carcinomas and matched normal mucosa tissues were constructed. The patients include 93 male and 87 female, and 62 patients of them diagnosed with distant metastasis. The clinicopathological features of the patients are summarized in Table 2. This research was approved by the Ethics Committee of Shanghai General Hospital and written informed consent was obtained from all patients.

Immunohistochemistry analysis was performed according to the standard protocol as previously described^36^. The stained slides were evaluated and scored independently by two pathologists (double blinded). The score was based on the extent and intensity of staining. Score the extent of staining was based on the percentage of positive tumor cells (0%, 0; 1–25%, 1; 26–50%, 2; 51–75%, 3; >75%, 4). Score of the intensity of staining (no staining, 0; weak staining, 1; moderate staining, 2; strong staining, 3). Overall score of each slide was calculated by this formula: overall score = extent score × intensity score. Then, a score of ≤6 was considered to be low expression; and a score of >6 was considered to be high expression. The primary antibodies involved in the immunohistochemistry analysis: RBBP6 (1:200, Novus, USA), p-p65 (1:100, Abcam, UK), N-cadherin (1:200, CST, USA), E-cadherin (1:400, CST, USA)

### Cell lines and cell culture

Human colon cancer cell lines (HT29, SW620, LoVo, Caco2, RKO, SW480, and HCT15) were obtained from American Type Culture Collection (ATCC, Manassas, VA, USA). All cells were cultured in Dulbecco’s modified Eagle’s medium with 10% fetal bovine serum (Gibco, CA, USA) at 37 °C in 5% CO_2_. Cell lines were authenticated using Short Tandem Repeat (STR) analysis and tested for mycoplasma contamination.

### Plasmid construction and retroviral transduction

For silencing RBBP6, the negative control shRNA (sh-NC) lentivirus and RBBP6 shRNA (sh-RBBP6) lentivirus were constructed by Genechem Biotechnology Company (Genechem, Shanghai, China). The target sequences for RBBP6 shRNA were as follows: sh-RBBP6-1, 5′-CCTTTGATGGGCTCCACAT-3′; sh-RBBP6-2, 5′-GATGACTCTTCCGCGTCTA-3′. HT29 and SW620 cells were transduced with 5 × 10^5^ TU/ml of lentivirus to construct HT29-sh-RBBP6 and SW620-sh-RBBP6 cells.

Lentiviral vectors harboring Cas9 and sgRNA (Genechem, Shanghai, China) for overexpressing RBBP6 were constructed. According to the manufacturer’s instructions, firstly, the lentiviral vectors harboring Cas9 were transfected into RKO and SW480 cells using polybrene, and the RKO-Cas9 or SW480-Cas9 stable cells were isolated. After that, the lentiviral vectors harboring sgRNA (sgRNA-RBBP6) were transfected into the RKO-Cas9 and SW480-Cas9 stable cells. Eventually, the Cas9-sgRNA-RBBP6 stable cell lines were established. We designed three different sgRNAs according to three target sites in RBBP6 gene, and the sequences of the sgRNAs were as follows: sgRNA-1386, AAGCGCGGGGTCACGTGGGC; sgRNA-1387, CTCTTTCAGTTTCCGCGCTT; sgRNA-1388, GGCTGACGTCACAAGCGCA.

HA-tagged full-length cDNA of RBBP6 (HA-RBBP6-full length), HA-tagged cDNA of RBBP6 lacking the RING Finger domain (HA-RBBP6-ΔRing Finger) and the region of RBBP6 encoding the RING Finger domain (HA-RBBP6-Ring Finger) were PCR-amplified and subcloned into pcDNA3.1 plasmids for transfection. pcDNA3.1-Flag-IκBα, pcDNA3.1-His-Ub, pcDNA3.1-p65, and pcDNA3.1-IκBα-Mutant (IκBα dominant-negative mutant, which can block NF-κB activation but cannot be ubiquitinated and degraded by proteasome) were also established.

### Cell viability assay

Cell viability was performed using the Cell Counting Kit-8 kit (Dojindo Laboratories, Japan) according to the manufacturer’s protocols. HT29, RKO, SW620, and SW480 cells were diluted, and then about 3 × 10^3^ cells were seeded into 96-well plate containing 100 μl of medium per well. Cell viability was detected at 12, 24, 48, 72, and 96 h by measuring the absorbance at 450 nm. All assays were performed in triplicate.

### Cell wound healing assay

Cells were cultured to full confluence in a six-cell plate. Then, a scratch was generated using a micropipette tip. Cells were washed by phosphate-buffered saline (PBS) and cultured with serum-free medium. The scratches were then photographed at 0, 12, and 24 h, and cell migration was compared by measuring the gap size in each field. The experiment was performed in triplicate.

### Transwell migration and invasion assays

Cell migration and invasion assays were determined using Transwell 24-well Boyden Chambers (BD Biosciences, USA). For invasion assays, the Corning Matrigel (Corning, USA) was thawed at 4 °C overnight. The Matrigel was then diluted in serum-free DMEM to the final concentration of 200 μg/mL. Next, about 100 μL of the diluted Matrigel was carefully added to the upper chamber and incubated at 37 °C for 30 min to allow the Matrigel dilution to form a gel. For migration assays, the upper chambers were not coated with Matrigel. After that, a total of 200 μL of the cell suspension containing about 1 × 10^5^ cells was added into the upper chamber of each Transwell. A total of 600 μL of medium containing 15% FBS was added to the lower chamber of each well. The cells were cultured at 37 °C with 5% CO_2_ in a humidified incubator. Cells that migrated or invaded to the reverse side of the upper chamber were stained and counted. Each experiment was repeated in triplicate.

### Western blot analysis

Total protein was isolated using RIPA solution (Beyotime Biotechnology, Jiangsu, China) and BCA protein assay kit (Beyotime Biotechnology, Jiangsu, China) was applied to detect the concentration of the protein. Equal amounts of the protein (50 μg) were spotted into 10% SDS–PAGE gel for electrophoresis, followed by transferring onto PVDF membranes. Then, the membranes were soaked in 5% milk for an hour at room temperature. Subsequently, membranes were incubated at 4 °C overnight with primary antibodies against RBBP6 (1:1000, Novus, USA), N-cadherin (1:1000, CST, USA), Vimentin (1:1000, CST, USA), Snail (1:1000, CST, USA), E-cadherin (1:1000, CST, USA), p65 (1:1000, CST, USA), IKKβ (1:1000, CST, USA), IκBα (1:1000, CST, USA), p-p65 (1:1000, CST, USA), p-IKKβ (1:500, CST, USA), p-IκBα (1:500, CST, USA), HA (1:1000, Santa Cruz, USA), Flag (1:1000, Santa Cruz, USA), His (1:1000, Santa Cruz, USA), Histone H3 (1:500, Abcam, UK), Tubulin (1:1000, CST, USA), and GAPDH (1:2000, Abcam, UK). After that, the membranes were washed by PBS and incubated with HRP-tagged secondary antibody for 1 h. The proteins were then detected using ECL reagent (Pierce Biotechnology, USA). GAPDH or Tubulin levels served as loading controls. All experiments were repeated in triplicate.

### Immunofluorescence staining

Cells were fixed with 4% paraformaldehyde for 15 min and permeabilized with 0.1% Triton X-100 for 5 min. After blocked in 5% normal goat serum for 30 min, the cells were incubated with primary antibodies at 4 °C overnight. Then the cells were stained by fluorophore-conjugated secondary antibody for 1 h and nuclei were counterstained with 4′,6-diamidino-2-phenylindole (DAPI) for 10 min in dark. Confocal immunofluorescence was performed by a Leica TCS SP8 confocal laser-scanning microscope (Leica, Wetzlar, Germany). The experiment was performed in triplicate.

### Co-immunoprecipitation

Total protein was extracted from HEK293 cells co-transfected with HA-RBBP6, Flag-IκBα, and His-Ub plasmids. Cell lysates were then incubated with 2 μg indicated antibodies at 4 °C overnight. Subsequently, the antibodies were pulled down with 25 µl protein A/G Agarose beads (Santa Cruz, USA) for 4 h at 4 °C. After that, the beads were separated and washed by PBS. Samples were then subjected to western blot analysis.

### Luciferase reporter assay

For the luciferase reporter assay of NF-κB, pGL6-TA plasmid containing NF-κB/p65 response element, and pRL-TK plasmid were co-transfected into the stable HT29 or RKO cells using Lipofectamine 2000. After transfection for 48 h, both firefly and renilla luciferase activities were detected by a dual luciferase reporter system according to the manufacturer’s protocol.

### In vivo tumorigenicity and metastasis assay

For tumorigenicity assay in vivo, HT29 or RKO cells (2 × 10^6^) were suspended in 150 μl of PBS and subcutaneously injected into the groins of the 4-week-old male nude mice (Institute of Zoology, Chinese Academy of Science, Shanghai, China). Tumor volumes and tumor weights were measured on the indicated days. After implantation for 30 days, the mice were sacrificed and the tumors were obtained for IHC staining and Western blot analysis. The tumor volume was calculated by this formula: volume (mm^3^) = (longer diameter × shorter diameter^2^)/2.

For metastasis assay in vivo, luciferase-labeled cells (5 × 10^6^) were injected into the tail vein or spleen of the mice to construct the lung metastasis model or the hepatic metastasis model, respectively. After implantation for 60 days, the lungs and livers were harvested for H&E staining. Then, the number and diameter of metastatic nodules were calculated in the tissue section. For luciferase imaging, we injected d-luciferin-potassium salt into the abdominal cavity of the mice. 7 min after the injection, mice were anesthetized and pictures were captured by an IVIS-100 system (Caliper Life Sciences, USA).

All the animal experiments were performed on the basis of Animal Care Guidelines of Shanghai General Hospital, Shanghai Jiao Tong University. Nude mice were divided into control group and experimental groups randomly. Each group contains five mice.

### Statistical analysis

All data were analyzed by SPSS 22.0 statistical software (SPSS, Chicago, IL, USA). The statistical significance between the clinicopathological features and protein expression levels was measured using the *χ*^2^ test. Survival curves were calculated with the Kaplan–Meier method and compared by the log-rank test. The Cox proportional hazard was applied to analyze the hazard risk and 95% confidence interval for DFS or OS. For all studies, *p*-value < 0.05 with a two-sided test indicates statistically significant.

### Ethics approval

All procedures performed in studies involving human participants were in accordance with the ethical standards of the Ethics Committee of Shanghai General Hospital, and with the 1964 Helsinki declaration and its later amendments. All participants provided informed written consent.

## Supplementary information

Figure S1

Figure S2

Figure S3

Figure S4

Figure S5

Figure S6

Supplementary Figures Legends

## Data Availability

The data that support the findings of this study are available from the corresponding author upon reasonable request.
