# Supplementary figures and images for: RBBP6, a RING finger-domain E3 ubiquitin ligase, induces epithelial–mesenchymal transition and promotes metastasis of colorectal cancer
Source: Cell Death Dis. 2019 Nov 4;10(11):833. doi: 10.1038/s41419-019-2070-7 (PMC6828677; doi:10.1038/s41419-019-2070-7)

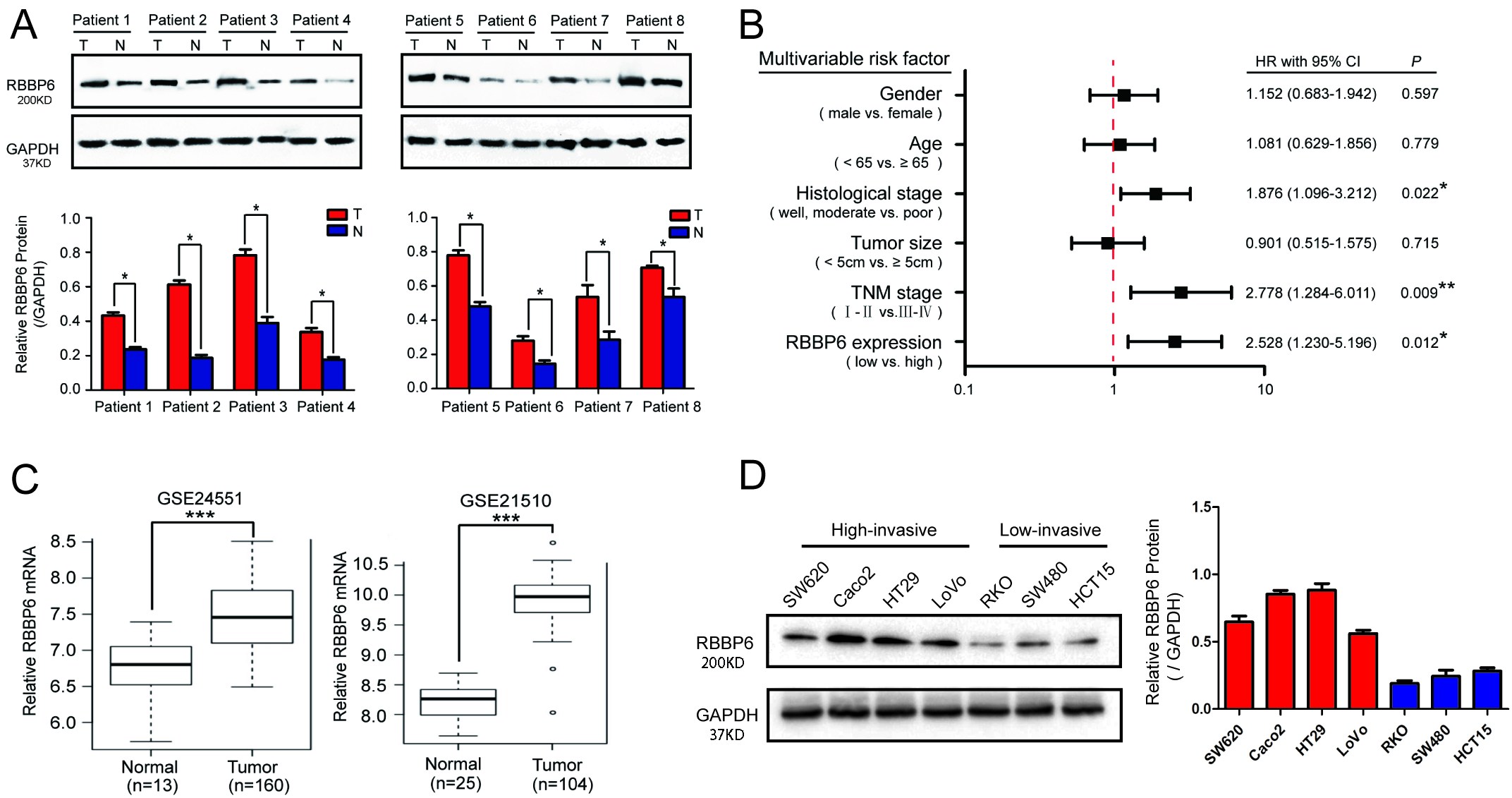

Supplement: Supplementary file 1 — Figure S1 [file 41419_2019_2070_MOESM1_ESM.tif]

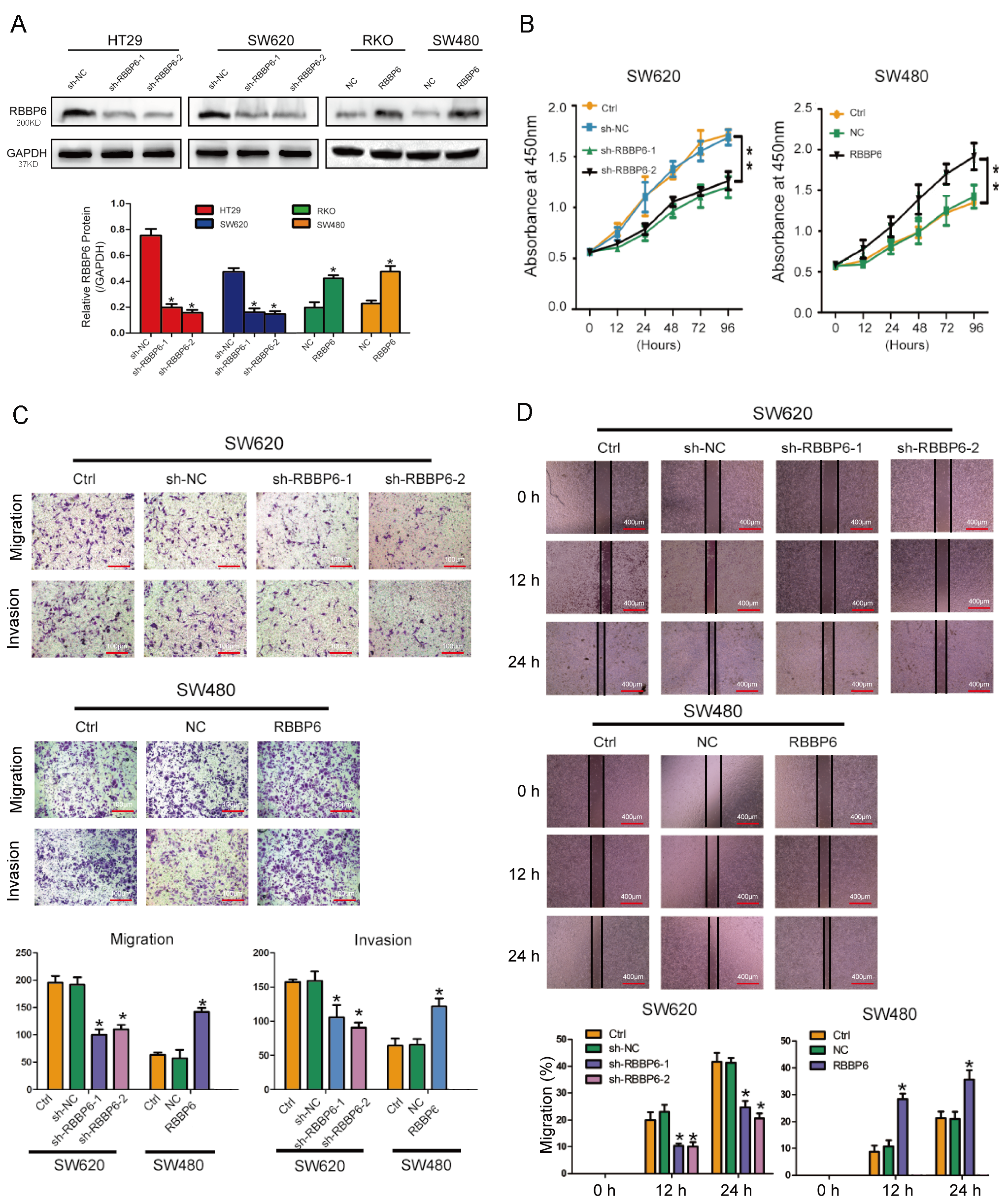

Supplement: Supplementary file 2 — Figure S2 [file 41419_2019_2070_MOESM2_ESM.tif]

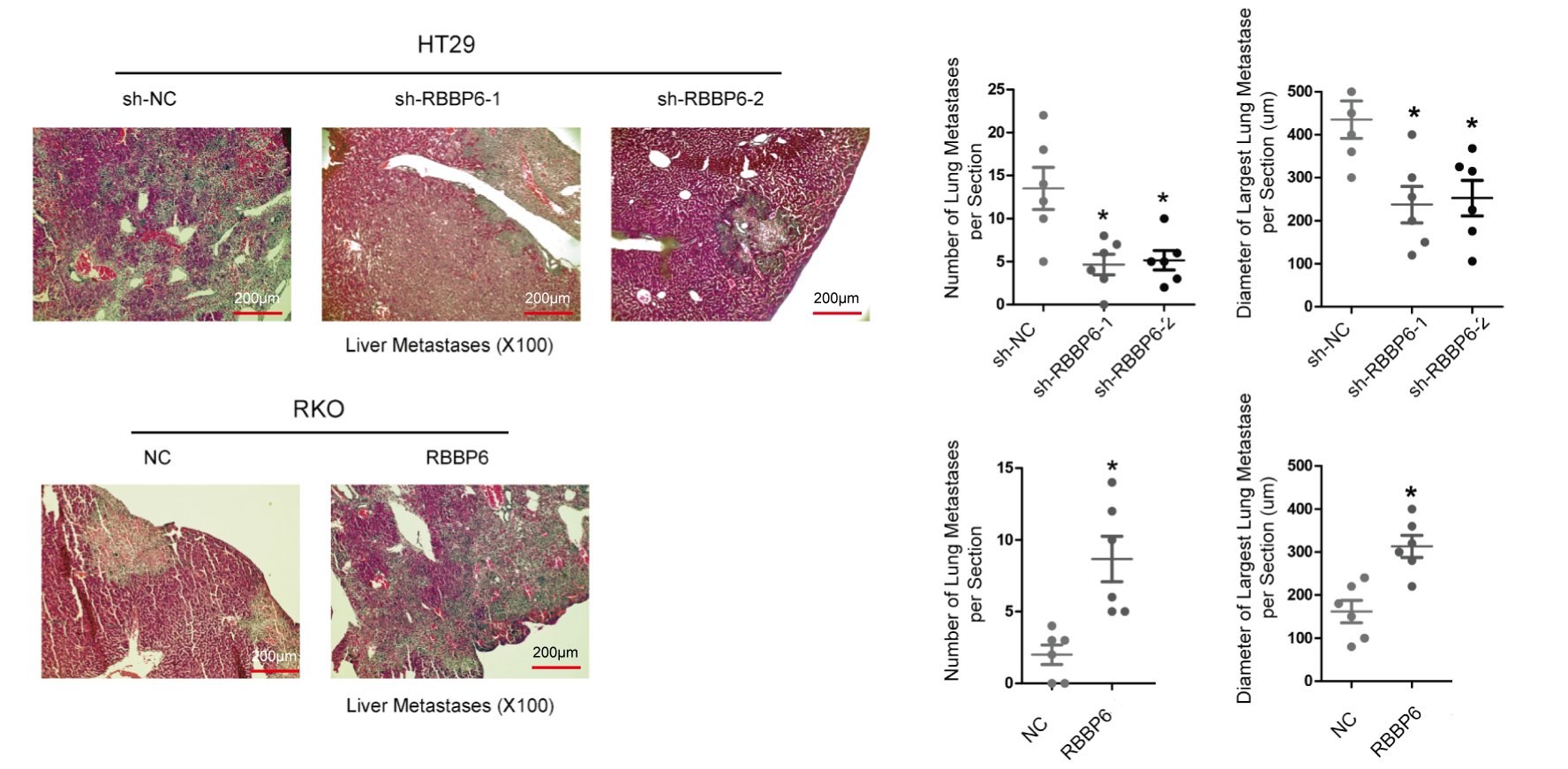

Supplement: Supplementary file 3 — Figure S3 [file 41419_2019_2070_MOESM3_ESM.tif]

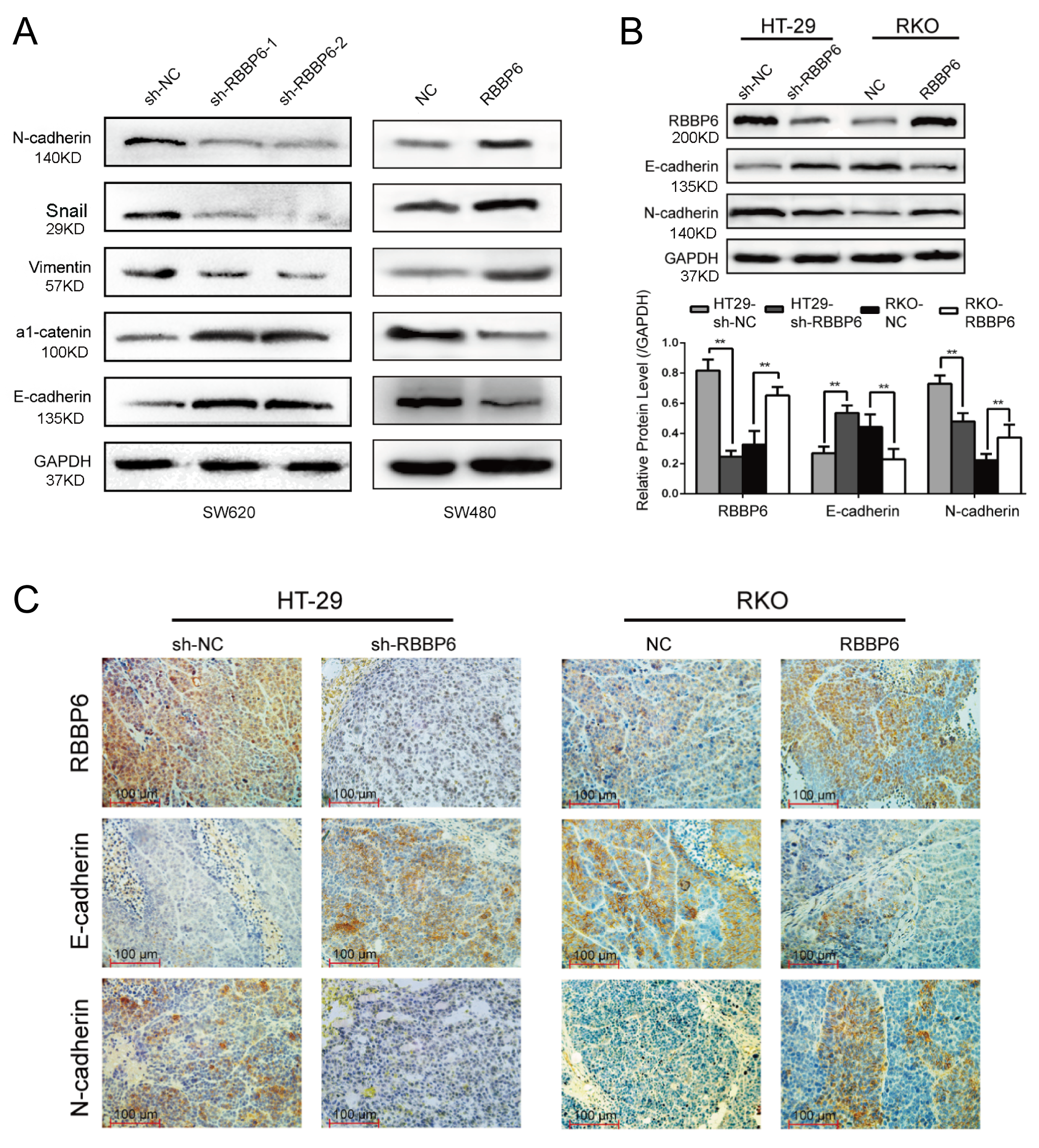

Supplement: Supplementary file 4 — Figure S4 [file 41419_2019_2070_MOESM4_ESM.tif]

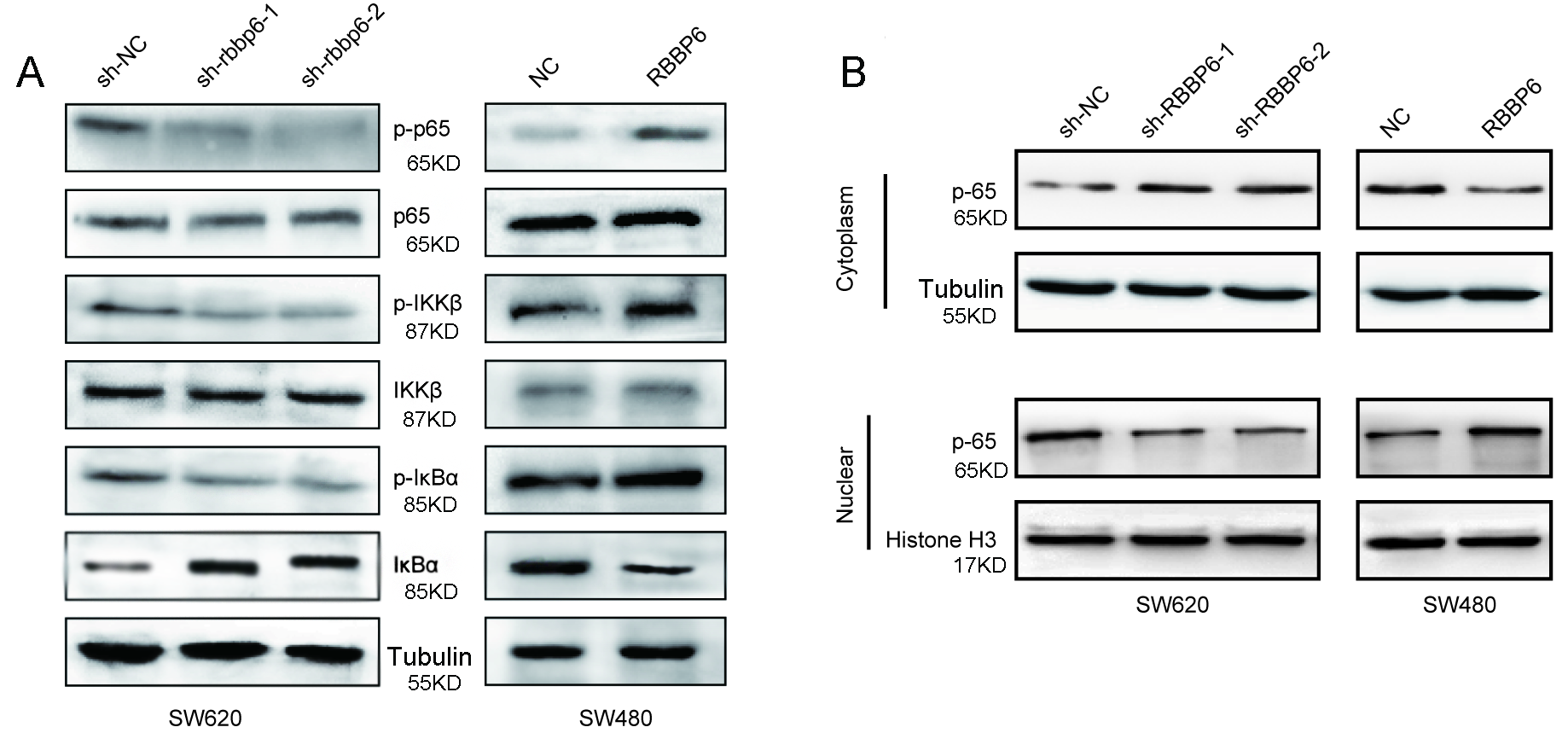

Supplement: Supplementary file 5 — Figure S5 [file 41419_2019_2070_MOESM5_ESM.tif]

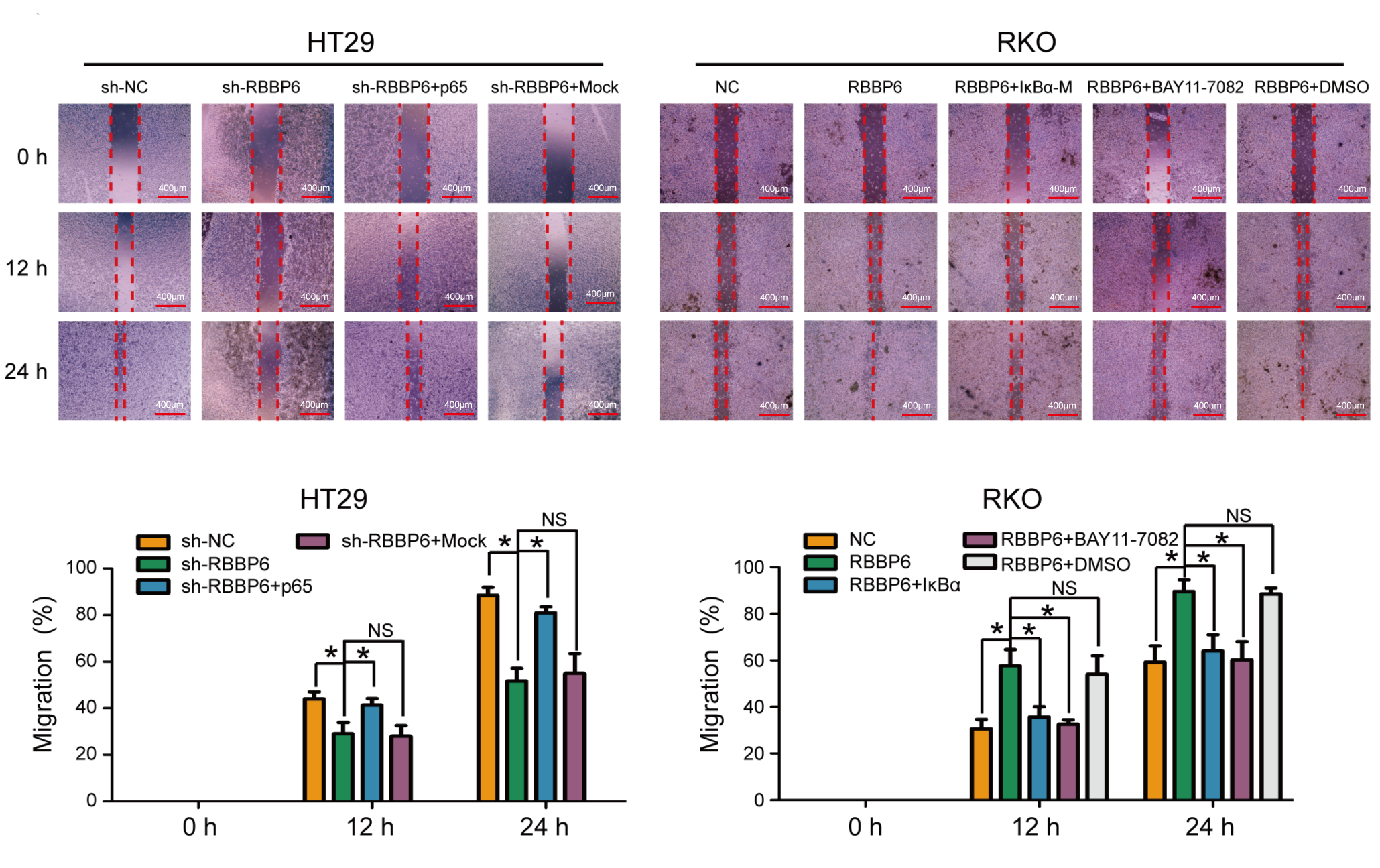

Supplement: Supplementary file 6 — Figure S6 [file 41419_2019_2070_MOESM6_ESM.tif]
