# Supplementary material for: RBBP6, a RING finger-domain E3 ubiquitin ligase, induces epithelial–mesenchymal transition and promotes metastasis of colorectal cancer
Source: Cell Death Dis. 2019 Nov 4;10(11):833. doi: 10.1038/s41419-019-2070-7 (PMC6828677; doi:10.1038/s41419-019-2070-7)
Supplement: Supplementary file 7 — Supplementary Figures Legends [file 41419_2019_2070_MOESM7_ESM.docx]

**Supplementary Figure Legends:**

**Supplementary Fig.1 RBBP6 expression and Multivariable analysis in CRC patients.**

**A**, Western blot analysis of RBBP6 expression in 8 representative cases of colorectal cancer specimen. **p* < 0.05. **B**, Expression of RBBP6 in normal specimens and colorectal carcinoma GEO datasets (GSE24551, GSE21510). **C**, Multivariable analysis was performed in the 180 patients, the bars correspond to 95% confidence intervals. **D**, Western blot analysis of RBBP6 expression in highly-invasive and low-invasive human CRC cells. **p* < 0.05.

**Supplementary Fig.2 RBBP6 promotes CRC cell proliferative, migratory and invasive capacity in SW620 and SW480 cells.**

**A,** The RBBP6 knockdown and overexpression effects were confirmed by Western blot. **B,** Cell proliferation rates were measured by CCK8 assay. The results were measured at an optical density of 450 nm. **C-D,** Cell motility were analyzed by wound healing assay, transwell migration and matrigel invasion assays. Data represent three independent experiments. Data are means ± SD. **p* < 0.05.

**Supplementary Fig.3** Hepatic metastasis model was constructed: Representative images of mice injected with stable cells and H&E staining of the metastatic nodules in the liver (left); The numbers and diameters of metastatic nodules in the livers of mice (right). Data are means ± SD. **p* < 0.05.

**Supplementary Fig.4 RBBP6 promotes CRC cell motility by modulating EMT.**

**A,** Expression of mesenchymal markers (N-cadherin, Snail and Vimentin) and epithelial markers (E-cadherin and α_1_-catenin) was analyzed by Western blotting in SW620 and SW480 cells. **B-C**, Expression of RBBP6, N-cadherin and E-cadherin in xenograft tumors was confirmed by Western blot and Immunohistochemistry.

**Supplementary Fig.5 RBBP6 promotes the activation of NF-κB signaling pathway in CRC.**

**A**, Proteins involved in the NF-κB signaling pathway were determined by Western blotting in SW620 and SW480 cells. **B**, Levels of p65 in nuclear and cytoplasmic extracts were measured by Western blotting in SW620 and SW480 cells.

**Supplementary Fig.6** Wound healing assay of RBBP6-knockdown HT29 cells transfected with plasmid expressing p65 (left); Wound healing assay of RBBP6-overexpression RKO cells treated with plasmid expressing IκBα-M or NF-κB inhibitor BAY11-7082 (right).
